# Incidence and predictors of paternal anxiety and depression following fetal abnormalities requiring pregnancy termination: a cross-sectional study in China

**DOI:** 10.1186/s12884-022-04739-3

**Published:** 2022-05-26

**Authors:** Shiwen Sun, Yuping Hao, Jialu Qian, Fang Wang, Yaping Sun, Xiaoyan Yu

**Affiliations:** 1 Department of Nursing, Women’s Hospital School of Medicine, Zhejiang University, Hangzhou, Zhejiang China; 2 Emergency Department, Tongji Hospital, Tongji Medical College of Huazhong University of Science and Technology, Wuhan, Hubei China; 3 Zhejiang University School of Medicine, Hangzhou, Zhejiang China

**Keywords:** Anxiety, Depression, Fathers, Stillbirth, Fetal abnormalities, Nursing

## Abstract

**Background:**

China is a country with a high prevalence of fetal abnormalities. Termination of pregnancy for fetal abnormalities (TOPFA) is a devastating traumatic event for parents and families, resulting in serious and lasting psychological problems. The impact of TOPFA on mothers has been extensively explored, but little research has been conducted on the resulting paternal psychological problems. This study sought to determine the prevalence and predictors of paternal anxiety and depression following TOPFA.

**Methods:**

We analysed cross-sectional data from 169 Chinese couples (169 mothers and 169 fathers) who experienced TOPFA. Anxiety was assessed with the Self-Rating Anxiety Scale (SAS), and depression was measured with the Self-Rating Depression Scale (SDS) for fathers and the Edinburgh Postnatal Depression Scale (EPDS) for mothers. We used the Social Support Rating Scale (SSRS) to assess levels of social support.

**Results:**

Overall, 19.5% of fathers and 24.3% of mothers had symptoms of anxiety, but there was no significant difference in the incidence of anxiety between fathers and mothers. However, depression was more common in mothers (50.3%) than in fathers (24.9%). Level of income (β = -2.945, 95% CI: -5.448 to -0.442), worry about the pregnancy (β = 3.404, 95% CI: 1.210 to 5.599) and objective support (β = -0.668, 95% CI: -1.163 to -0.173) were predictors of anxiety in fathers. Worry about the pregnancy (β = 4.022, 95% CI: 1.630 to 6.414), objective support (β = -0.652, 95% CI: -1.229 to -0.075) and maternal depression (β = 0.497, 95% CI: 0.159 to 0.836) were predictors of paternal depression.

**Conclusion:**

Anxiety and depression were prevalent among parents following TOPFA in China, and fathers had similar levels of anxiety as mothers. Strategies to support fathers should consider social support and psychological interaction and draw upon father-inclusive intervention recommendations.

## Background

With the development of prenatal diagnostic technology, the improvement of prenatal health care and the increase in age or high-risk status of pregnant women, the detection rate of fetal abnormalities is correspondingly on the rise [1, 2]. According to the WHO, in 2017, 280,000 newborns worldwide died of congenital malformations within 28 days of birth [3]. In China, approximately 1 million infants are born with birth defects every year, accounting for 5.6% of total births; the neonatal mortality rate caused by birth defects is increasing every year [4].

Fetal abnormality is an intense traumatic event that can cause psychological crises and complex psychological problems in parents, including anxiety, depression, posttraumatic stress disorder (PTSD), complicated grief and even suicidal thoughts [5–8]. More than half of parents still experience these symptoms at least four years after fetal loss [9]. The impact of perinatal loss on mothers has been extensively explored, but its influence on fathers is often neglected [6]. Traditionally, fathers are mainly thought to play the role of supporters; however, fathers also experience high levels of grief after pregnancy loss, which often goes unacknowledged by family, friends and society [6, 10]. Many fathers do not feel that they are accepted as legitimately grieving parents [8], which may lead to the suppression of sadness, anxiety, and stress, potentially increasing the risk of chronic psychological problems [8]; moreover, fathers may experience more anxiety in subsequent pregnancies [11]. In addition, divorce and/or marital problems after stillbirth are often reported. For some couples, differences in response to grief can lead to arguments, infidelity, and even domestic violence or suicidal tendencies [8]. In terms of sex differences in psychological disorders, some studies have shown that fathers also suffer from high proportions of anxiety and depression (16.5% and 24.4%, respectively), although the incidence of anxiety and depression in fathers is lower than that in mothers [12].

A review revealed that marital status, social support, negative appraisals, and variables correlated with care and management after stillbirth affected symptoms of anxiety and depression in parents [7]. Of the above factors, being unmarried was related to the highest risk of depression, and the second highest risk factor was dissatisfaction with the emotional support received. Kokou-Kpolou et al. [13] found that different negative thoughts are related to persistent depressive symptoms following perinatal loss and vary with the type of loss. In addition, care and management after stillbirth, including seeing/holding the stillborn baby, having tokens of remembrance, the degree of sharing memories and the attitudes and behaviors of the care providers, also affected parental psychological symptoms [7, 12, 14, 15]. If parents were not allowed to be with the stillborn baby for as long as they desired, it was a significant risk factor for parental depressive and anxiety symptoms [16, 17]. Furthermore, some researchers examined the mutual influence between psychological disorders and found that higher levels of parental grief were correlated with higher levels of both depression and anxiety [18]. Esra et al. [5] also confirmed that the stress levels of mothers increased as the stress levels of fathers increased, and vice versa.

Few studies have focused on the prevalence and predictive factors of anxiety and depression in fathers who experienced TOPFA. In this cross-sectional study, we sought to determine the prevalence of anxiety and depression in fathers and analyse sex differences and the correlation between parental anxiety and depression. In addition, we explored the factors that predicted paternal anxiety and depression.

## Methods

### Study design and setting

A cross-sectional survey was performed in the Women’s Hospital School of Medicine, Zhejiang University from November 2016 to October 2020. Convenience sampling was used to recruit fathers who experienced the loss of a baby either due to fetal abnormalities or stillbirth. Their wives were also recruited to the study. The inclusion criteria were as follows: participants experienced diagnosis of fetal abnormality or stillbirth confirmed by Zhejiang Provincial Prenatal Diagnosis Center and decided to terminate the pregnancy due to fetal abnormality or stillbirth after > 12 gestational weeks. The recruited couples were required to be older than 18 years of age and capable of fluent verbal communication. All participants were voluntary participation in the study and gave written informed consent prior to participation. The exclusion criteria included the following: a history of psychosomatic disease or any other traumatic event (bereavement, traffic accident, earthquake, or flood); intellectual disability or illiteracy; and/or an inability to understand the content of the questionnaires. According to the Kendall sample calculation method for studies exploring predictive factors, the sample size should be at least 5–10 times greater than the number of variables [19]. The maximum number of questionnaire items used in this study was 20; thus, the sample size required for the questionnaire survey was determined to be 100–200.

### Data collection

This study was approved by the ethics committee of the Women’s Hospital School of Medicine, Zhejiang University (IRB No. 20150071). Trained researchers collected data by face-to-face data collection methods. Participants signed informed consent forms and completed the personal information form and self‐report questionnaire independently after the researchers explained the aim and procedure of the study to them on the day of admission. All collected data remained confidential. The completeness of the questionnaires was further checked by the researchers on a daily basis. Of the 400 questionnaires sent out, 169 couples (338 participants) both completed it, a response rate of 84.5%, the remaining participants refused to participate in this study or did not complete the questionnaire due to lack of time or unwillingness to participate.

### Measurement instruments

The personal information form was created for this study and consisted of two parts as follows: demographic characteristics (including age, education level, employment status, religious beliefs, level of income, residential area, and health insurance) and perinatal loss characteristics (including gestational week, manner of fertilization, abnormal pregnancy history, diagnosis of fetal abnormalities, paternal knowledge, worries and expectations of their wife’s pregnancy).

#### Self-Rating Anxiety Scale (SAS)

The Self-Rating Anxiety Scale (SAS), which was developed by Zung [20] in 1971, was used to measure parent’s anxiety level. This scale consists of 20 items and each item was answered with “no or seldom,” “occasionally,” “usually,” “always.” For scoring of the answer, items 5, 9, 13, 17, and 19 were positive rated on a 4–1 scale whereas others were negative rated on a 1–4 scale. The scores of each item are added to obtain the rough score, which is multiplied by 1.25 and rounded to an integer to give the standard score. Standard scores < 50, 50–59, 60–69, and ≥ 70 represent no anxiety symptoms, mild anxiety, moderate anxiety and severe anxiety, respectively. Cronbach’s α of the scale is 0.852 [21].

#### Self-Rating Depression Scale (SDS)

Paternal depression was measured by the Self-Rating Depression Scale (SDS), a 20-item scale created by Zung [22] in 1965. The SDS is widely used to assess depression symptoms. It uses a 4-point Likert scale to assess the frequencies of symptoms in the past 7 days, and it has 10 items that are scored in reverse. The scores of each item are added to obtain the rough score. The rough score is multiplied by 1.25 and rounded to an integer, which is the standard score. The standard SDS score can be used to categorize individuals into four categories [23]: no depression (≤ 52), mild depression (53–62), moderate depression (63–72) and severe depression (≥ 73). Cronbach’s α of the SDS is 0.895 [21].

#### Edinburgh Postnatal Depression Scale (EPDS)

The Edinburgh Postnatal Depression Scale (EPDS), which is a 10-item scale on a 4-point scale, was used to measure maternal depression. It is widely used to screen women for depression symptoms in the perinatal period [24]. The EPDS was adapted to Chinese and checked for validity and reliability by Lee et al. [25] in 1998. Total scores ≥ 13 meet the diagnostic criteria for depression [24]. The specificity and sensitivity of EPDS have been evaluated in various international studies that have explored both minor and major depression. Cronbach’s α for this scale is 0.901 [26].

#### Social Support Rating Scale (SSRS)

We used the Chinese version of the self-report Social Support Rating Scale (SSRS) [27] to assess couples’ social support. The SSRS, which has been widely used to evaluate the social support levels of various populations, comprises three subscales: objective support (Items 2, 6, and 7), subjective support (Items 1, 3, 4, and 5), and availability of support (Items 8–10). Total scores of < 35, 35–45, and > 45 represent low, moderate, and strong social support, respectively. The SSRS exhibits good reliability and validity. The retest reliability r of this scale is 0.92, and Cronbach's α is 0.89–0.94 [28].

### Statistical analysis

All data analyses were performed using SPSS software (version 20.0; SPSS Inc., Chicago, IL, USA). Continuous variables are shown as means with SD; categorical variables are reported as percentages. Correlations were evaluated using Spearman’s correlation analyses, psychological outcomes were compared between sexes using the chi-square test and a suitable post hoc test (eg. z-tests) for categorical data. The continuous data were analysed by Mann–Whitney U test for comparison of two groups and by Kruskal Wallis test for multiple groups. A multivariate linear regression analysis was used to identify factors correlated with paternal anxiety and depression. *P* values < 0.05 were considered statistically significant.

## Results

### Participant characteristics

169 couples (169 fathers and 169 mothers) were included in this study. The perinatal loss information was as follows: the gestational week of fetal abnormality diagnosis was 26.71 ± 6.16 weeks; manner of fertilization: 9 (5.3%) had assisted reproduction, and the remainder conceived naturally; 24 (14.2%) had an abnormal pregnancy history. The top three categories of fetal diagnosis in the sample were as follows: 17.8% urinary system abnormalities, 16.0% neurological abnormalities, 16.0% cardiac abnormalities, and 14.2% multisystem abnormalities (Table 1). The demographic and perinatal loss characteristics of fathers are presented in Table 2.

**Table 1:** Fetal diagnosis

	***N* = 169(%)**
Urinary system abnormalities	30 (17.8)
Neurological abnormalities	27 (16.0)
Cardiac abnormalities	27 (16.0)
Multisystem abnormalities	24 (14.2)
Chromosomal or gene abnormalities	17 (10.0)
Stillbirth	14 (8.3)
Facial abnormalities	11 (6.5)
Skeletal abnormalities	6 (3.6)
Digestive system abnormalities	4 (2.4)
Other abnormalities	9(5.3)

**Table 2:** Demographic and perinatal loss characteristics of the participants and difference in the SAS and SDS scores disaggregated by various characteristics (*N* = 169)

Demographic Characteristics	N	SAS mean ± SD	*P* value	SAS mean ± SD	*P* value
Age					
18–29	73	42.32 ± 9.05	0.409	46.12 ± 11.09	0.713
30–39	76	40.83 ± 8.87		44.72 ± 11.71	
≥ 40	20	40.70 ± 14.84		44.95 ± 14.50	
Education Level					
Junior high school/below	29	44.83 ± 10.61	0.001*	49.00 ± 11.06	0.000**
Senior high school	41	43.37 ± 8.71		48.39 ± 12.02	
Junior college	43	42.60 ± 10.46		46.74 ± 11.75	
Bachelor’s degree/above	56	37.43 ± 8.36		40.18 ± 10.33	
Employment status					
Employed	157	41.32 ± 9.48	0.859	45.50 ± 11.89	0.594
Unemployed	12	43.25 ± 13.55		43.42 ± 10.04	
Religious belief					
No belief	140	40.98 ± 9.95	0.103	44.72 ± 11.85	0.136
Had beliefs	29	43.76 ± 8.73		48.41 ± 10.96	
Level of income					
Low	12	47.33 ± 11.55	0.001*	46.42 ± 13.29	0.014*
Medium	60	43.93 ± 8.81		48.72 ± 11.30	
High	97	39.20 ± 9.54		43.14 ± 11.45	
Residential area					
City	76	40.41 ± 9.32	0.467	43.70 ± 11.60	0.270
Towns	48	42.25 ± 10.70		46.83 ± 12.70	
Villages	45	42.38 ± 9.56		46.58 ± 10.83	
Health insurance					
Yes	116	40.31 ± 9.19	0.050*	44.27 ± 11.23	0.141
No	53	43.96 ± 10.63		47.74 ± 12.62	
**Perinatal loss characteristics**					
Knowledge of pregnancy					
Know well	23	43.48 ± 12.72	0.109	44.65 ± 13.79	0.119
A little understanding	124	40.45 ± 9.08		44.66 ± 11.46	
Incomprehension	22	45.00 ± 9.46		50.00 ± 10.53	
Worries about pregnancy					
No worry	22	36.00 ± 7.38	0.000**	36.86 ± 9.27	0.000**
A little worried	73	39.25 ± 8.79		44.26 ± 11.06	
Extremely worry	74	45.26 ± 9.99		48.96 ± 11.69	
Expectations of pregnancy					
A little anticipation	44	37.68 ± 8.51	0.002*	42.93 ± 10.85	0.161
Extremely anticipation	125	42.78 ± 9.88		46.21 ± 11.98	

^*^P < 0.05

**P < 0.001

### Anxiety, depression and social support

The anxiety scores of fathers and mothers that had experienced fetal abnormalities were 41.46 ± 9.78 and 43.07 ± 10.17, respectively. The incidence of anxiety in fathers and mothers was 19.5% (33/169) and 24.3% (41/169), respectively. There was no significant difference in the incidence of anxiety between fathers and mothers (χ^2^ = 1.107, *P* = 0.293, Cramer’s V = 0.057). The SDS score of fathers was 45.36 ± 11.76, and the incidence of depression was 24.9% (42/169). The EPDS score of mothers was 12.67 ± 4.76, and the incidence of depression was 50.3% (85/169). The incidence of maternal depression was significantly higher than that of fathers (*χ*^2^ = 23.322, *P* < 0.001, Cramer’s V = 0.263). The fathers’ social support score was 42.47 ± 6.33, and the proportions of good, moderate and poor social support were 29.0% (49/169), 59.2% (100/169) and 11.8% (20/169), respectively.

### Association between anxiety, depression and social support

Correlation analyses showed that the father’s SAS scores had a low positive correlation with those of the mothers (*r* = 0.177, *P* = 0.021) and a moderate negative correlation with fathers’ SSRS scores (*r* = -0.292, *P* < 0.001). Similarly, fathers’ SDS scores were moderately positively correlated with the EPDS scores of the mothers (*r* = 0.299, *P* < 0.001) and moderately negatively correlated with the father’s SSRS scores (*r* = -0.373, *P* < 0.001). Table 3 showed the associations between the scores of all SSRS dimensions and fathers’ SAS scores and SDS scores.

**Table 3:** The correlation between anxiety, depression and social support in fathers and mothers with fetal abnormalities

	**Fathers’ SAS**		**Fathers’ SDS**	
	**r**	**p**	**r**	**p**
Fathers’ SSRS	-0.292	< 0.001	-0.373	< 0.001
Objective support	-0.329	< 0.001	-0.369	< 0.001
Subjective support	-0.191	0.013	-0.240	0.002
Availability of support	-0.152	0.048	-0.248	0.001
Mothers’ SAS	0.177	0.021	0.193	0.012
Mothers’ EPDS	0.224	0.003	0.299	< 0.001

### Bivariate association among paternal anxiety and depression and demographic and perinatal loss characteristics

Table 2 provides bivariate associations among the study variables and paternal anxiety and depression. Univariate analyses indicated that five factors were associated with paternal anxiety: education level, level of income, worries and expectations of pregnancy, and health insurance (*p* ≤ 0.05). In order to avoid omission, there was no significant difference in univariate analyses, while multifactor analysis might be significant for influencing factors, therefore, this variable (health insurance) was included in the multivariate analysis. Additionally, three factors were associated with paternal depression: education level, level of income and worries about pregnancy.

### Multifactorial analysis of paternal anxiety and depression

Multivariate linear regression analyses were conducted with paternal anxiety and depression as the dependent variables and the aforementioned significantly associated factors as independent variables. In addition, there was no multicollinearity between all independent variables in the two regression models (VIF < 5). The results suggested that level of income and objective support were negatively associated with paternal anxiety; in contrast, worries about pregnancy were positively associated with paternal anxiety (see Table 4). Regarding paternal depression, worries about pregnancy and the depression level of mothers were important risk factors that could significantly increase paternal depression, while objective support was a protective factor against paternal depression (see Table 5).

**Table 4:** Multivariate linear regression analyses predicting anxiety of fathers (*n* = 169)

Variable	B	Beta	t	*P*	B 95% CI	
					**Lower**	**Upper**
Constant	42.890	–	5.384	< 0.001	27.155	58.624
Education Level	-0.311	-0.035	-0.390	0.697	-1.885	1.263
Level of income	-2.945	-0.189	-2.324	0.021*	-5.448	-0.442
Health insurance	0.497	0.024	0.301	0.764	-2.764	3.757
Worries about pregnancy	3.404	0.240	3.064	0.003*	1.210	5.599
Expectations of pregnancy	2.613	0.118	1.592	0.113	-0.628	5.853
Objective support	-0.668	-0.209	-2.666	0.008*	-1.163	-0.173
Subjective support	0.023	0.009	-0.124	0.901	0.336	0.381
Availability of support	-0.083	-0.015	-0.198	0.843	-0.905	0.740
Anxiety of mother	0.026	0.027	0.365	0.716	-0.116	0.168

*R*^2^ = 0.240; Adjusted *R*^2^ = 0.196; Durbin-Watson = 2.164; F = 5.564, *P* < 0.001

^*^P < 0.05

^**^P < 0.001

**Table 5:** Multivariate linear regression analyses predicting depression of fathers (*n* = 169)

Variable	B	Beta	t	*P*	B 95% CI	
					**Lower**	**Upper**
Constant	52.586	–	6.517	< 0.001	36.651	68.520
Education Level	-1.090	-0.102	-1.213	0.227	-2.865	0.684
Level of income	-0.040	-0.002	-0.028	0.978	-2.904	2.823
Worries about pregnancy	4.022	0.236	3.320	0.001*	1.630	6.414
Objective support	-0.652	-0.170	-2.232	0.027*	-1.229	-0.075
Subjective support	0.286	-0.098	-1.345	0.180	-0.706	0.134
Availability of support	-0.775	-0.115	-1.596	0.112	-1.734	0.184
Depression of mother	0.497	0.201	2.899	0.004*	0.159	0.836

*R*^2^ = 0.267; Adjusted *R*^2^ = 0.235; Durbin-Watson = 2.032; F = 8.383, *P* < 0.001

^*^P < 0.05

^**^P < 0.001

## Discussion

To the best of our knowledge, the present study is the first to investigate the prevalence and predictive factors of anxiety and depression among fathers who experienced TOPFA in China. Our results showed that 19.5% of fathers reported symptoms of anxiety, and mothers experienced a similar incidence of anxiety (24.3%). However, the prevalence of depression was significantly higher in mothers than in fathers (50.3% vs. 24.9%). Paternal social support level was mainly at the middle level. We also investigated the potential psychological association between fathers and mothers and found that paternal anxiety and depression were positively correlated with maternal anxiety and depression. Furthermore, maternal depression was an independent risk factor for paternal depression after adjusting for confounding variables. Worries about the pregnancy were a risk factor for paternal anxiety and depression, whereas social support was an important protective factor. In addition, the level of income was negatively associated with paternal anxiety.

In the present study, the prevalence of anxiety and depression in both mothers and fathers was inconsistent with those of previous studies [29]. Hennegan et al. [12] found that the prevalence of anxiety and depression in bereaved mothers was high (38.7% and 42.6%, respectively); similarly, high proportions of anxiety and depression were reported for fathers (16.5% and 24.4%, respectively). Sarkar et al. [30] found that 18.1% of fathers and 39.3% of mothers had depression symptoms. The inconsistency in the prevalence of anxiety and depression may partly be explained by the inconsistency of measurement tools and time since stillbirth that data were collected. When using the same scale and diagnostic criteria, the results of previous studies and ours were similar [31, 32]. Another explanation could be cultural differences, which may lead to differences in cognitive and psychological responses to TOPFA.

Consistent with international studies [12, 30, 33], we reported that maternal depression was more prevalent than that of paternal depression after TOPFA. However, maternal anxiety prevalence was similar to that of paternal anxiety. Other studies in the literature have shown that anxiety is more prevalent in mothers than in fathers after TOPFA [12, 30, 33]. A possible explanation for the difference might be that in traditional Chinese culture, fathers who have lost infants face pressure to carry on the family line and try to suppress their own negative emotions to support their bereaved spouses, thus potentially increasing symptoms of anxiety and the risk of chronic psychological problems.

We found that fathers’ psychological disorders, including anxiety and depression, were positively correlated with those of mothers. In particular, maternal depression was an independent risk factor for paternal depression. These results are novel findings. They are consistent with family system theory [34], which emphasizes interaction patterns in the family system. In this theory, the behavior of one family member affects the behavioral, cognitive and emotional changes of other members. Another study also confirmed that maternal stress levels increase as paternal stress levels increase, and vice versa [5], our study supports that conclusion.

The present study provides new insight into fathers’ pregnancy worries in abnormal pregnancies. Worries about pregnancy are a risk factor for paternal anxiety and depression. In the literature review, no studies referred to fathers’ worries about pregnancy in abnormal pregnancies, although fathers play an increasingly important role in the pregnancy and childbirth process of their wives [5, 35], including prenatal examinations, transformation into the roles of expectant parents, and care for the newborns. Especially in traditional Chinese culture, pregnancy is an event that affects the psychology of the whole family. Fathers may have various worries about pregnancy due to the lack of knowledge about pregnancy, especially fetal abnormalities, and thus have higher rates of anxiety and depression.

In our study, there were negative associations between paternal anxiety/depression and social support, which is consistent with previous studies [7, 36]. Support from health care providers and especially from family members, can significantly reduce anxiety and depression in perinatal bereaved parents [7].

Higher levels of income were a significant factor that protected fathers from developing anxiety. In the review, few studies were found regarding the factors affecting paternal anxiety after TOPFA. A Chinese survey in spouses of pregnant women with normal pregnancies showed that prenatal anxiety is more common among spouses with lower income levels [35]. Esra et al. [5] reported that the posttraumatic stress levels of fathers with higher income levels were lower than those of fathers with lower income levels. Those previous results are consistent with our findings. One explanation for this finding is that fathers with high income levels have good material security to reduce worries about the cost of treatment and rehabilitation for their wives. Additionally, they may pay more attention to mental health and be more active in seeking support to meet their psychological needs [31].

There were some limitations to the present study. First, the present study used a cross-sectional design, therefore, we could not determine causality. Second, the non-probabilistic convenience sampling method employed could limit the generalizability of the findings, and the study sample was small and recruited from a single hospital, which may have biased the analyses. Third, we used a self-report questionnaire rather than objective measures, so we cannot exclude the possibility of recall bias and social expectations. It is possible that fathers might have been reluctant to report their true psychological reactions because of social expectations. In addition, the included factors that influence anxiety and depression in this study were limited; future research should include contact with stillborn infants, adult personality characteristics, marital relationships, coping styles and other factors.

## Conclusions

Anxiety and depression are prevalent in fathers and mothers who experience TOPFA. Worry about pregnancy was a risk factor for anxiety and depression in fathers, whereas social support was an important protective factor. In addition, a negative psychological reaction in either the father or the mother affected the other member of the couple. These results emphasize that psychological interventions for pregnant women with fetal abnormalities should consider the psychological adjustment and recovery of both the mother and father. The factors affecting paternal anxiety and depression identified in this study should be considered when developing psychological interventions for parents. Medical staff should offer professional informational support and psychoeducation to parents to reduce fathers’ worries about pregnancy and improve social support for them as well as instructing them to make full use of social support.

## Data Availability

The datasets generated and/or analysed during the current study are not publicly available as ethical approval was not sought for their dissemination but are available from the corresponding author on reasonable request.

## Abbreviations

TOPFA: Termination of pregnancy for fetal abnormalities
SAS: Self-Rating Anxiety Scale
SDS: Self-Rating Depression Scale
EPDS: Edinburgh Postnatal Depression Scale
SSRS: Social Support Rating Scale
PTSD: Posttraumatic stress disorder
